# A Novel *SMAD4* Mutation Causing Severe Juvenile Polyposis Syndrome with Protein Losing Enteropathy, Immunodeficiency, and Hereditary Haemorrhagic Telangiectasia

**DOI:** 10.1155/2015/140616

**Published:** 2015-02-01

**Authors:** Joel Johansson, Christofer Sahin, Rebecka Pestoff, Simone Ignatova, Pia Forsberg, Anders Edsjö, Mattias Ekstedt, Marie Stenmark Askmalm

**Affiliations:** ^1^Gastroenterology and Hepatology Unit, Department of Clinical and Experimental Medicine, Faculty of Health Sciences, Linköping University, County Council of Östergötland, 581 85 Linköping, Sweden; ^2^Department of Clinical Pathology and Clinical Genetics and Department of Clinical and Experimental Medicine, Faculty of Health Sciences, Linköping University, County Council of Östergötland, 581 85 Linköping, Sweden; ^3^Division of Infectious Diseases, Department of Clinical and Experimental Medicine, Faculty of Health Sciences, Linköping University, County Council of Östergötland, 581 85 Linköping, Sweden; ^4^Department of Clinical Pathology and Genetics, Sahlgrenska University Hospital, 413 45 Gothenburg, Sweden

## Abstract

Juvenile polyposis syndrome (JPS) is a rare genetic disorder characterized by juvenile polyps of the gastrointestinal tract. We present a new pathogenic mutation of the *SMAD4* gene and illustrate the need for a multidisciplinary health care approach to facilitate the correct diagnosis. The patient, a 47-year-old Caucasian woman, was diagnosed with anaemia at the age of 12. During the following 30 years, she developed numerous gastrointestinal polyps. The patient underwent several operations, and suffered chronic abdominal pain, malnutrition, and multiple infections. Screening of the *SMAD4* gene revealed a novel, disease-causing mutation. In 2012, the patient suffered hypoalbuminemia and a large polyp in the small bowel was found. Gamma globulin was given but the patient responded with fever and influenza-like symptoms and refused more treatment. The patient underwent surgery in 2014 and made an uneventful recovery. At follow-up two months later albumin was 38 g/L and IgG was 6.9 g/L. Accurate diagnosis is essential for medical care. For patients with complex symptomatology, often with rare diseases, this is best provided by multidisciplinary teams including representatives from clinical genetics. Patients with a *SMAD4* mutation should be followed up both for JPS and haemorrhagic hereditary telangiectasia and may develop protein loosing enteropathy and immunodeficiency.

## 1. Introduction

Juvenile polyposis syndrome (JPS) is a rare genetic disorder characterized by juvenile polyps of the gastrointestinal tract; additional extra intestinal manifestations include telangiectasias. This disorder is most frequently caused by mutations of the* SMAD4* or* BMPR1A* genes, although mutations of a variety of genes have been known to cause this syndrome. Most of the genes are associated with the BMP/TGF-*β* signalling pathways [1]. JPS is a clinical diagnosis where one of three criteria must be met: ≥5 juvenile polyps in the large intestine, multiple juvenile polyps throughout the gastrointestinal tract, or any number of juvenile polyps in a person with family history of juvenile polyposis [2].

*SMAD4* mutations have been known to cause JPS complicated with both haemorrhagic hereditary telangiectasia (HHT) [3] and protein loosing enteropathy (PLE) [4]. The diagnosis of HHT depends upon fulfilling the Curaçao criteria [5].

We here present a novel* SMAD4* mutation causing JPS and HHT. The report illustrates the need of multidisciplinary health care teams and that adequate genetic assessment has to be performed in order to facilitate the correct diagnosis.

## 2. Case Report

At the age of 12 the patient was sent to the school nurse by her teacher due to paleness. The diagnosis of anaemia was made and she was referred to the paediatric clinic in Linköping, Sweden, where her anaemia was attributed to frequent episodes of epistaxis.

During the next 20 years, the patient developed a large number of gastrointestinal polyps that often presented with bleeding in the colon, stomach, or small intestine. She underwent several operations including a total colectomy in 1984, gastrectomy (Roux-en-Y) due to polyps, and hysterectomy in 1995 due to bleeding. At a later stage, the patient suffered from intestinal obstruction with ischemia and 20 cm of the bowel was resected in 1999. As part of the preoperative work-up before the hysterectomy, prothrombin time (PT), thrombocyte particle count (TPK), and activated partial thromboplastin time (APTT) were normal. Although polyps were noted already in 1984, no definitive diagnosis was made. Several diseases were suspected including Peutz-Jeghers syndrome, coeliac disease, amyloidosis, and familial adenomatous polyposis (FAP).

From the early 90s chronic abdominal pain was a large part of the patient's symptomatology along with diarrhea. After the gastrectomy, the abdominal pain got worse, especially with food intake. Total parenteral nutrition was implemented but had to be cancelled due to multiple infections and sepsis with coagulase negative staphylococci. Venofer injections were tried but the effect was poor and, at times, so was compliance. The patient lost a lot of weight and albumin levels were frequently low.

In the beginning of the 21st century, the patient's health deteriorated and she suffered dental problems and had to extract all of her teeth. She was admitted several times for sepsis, pneumonia, and drug poisoning and slowly her treating physicians realized that she suffered from an iatrogenic mixed substance abuse, mainly opioid analgesics and benzodiazepines. In 2005, bilateral pitting oedema developed and laboratory results from the same time period showed hypoalbuminemia, with an albumin of 26 g/L, and iron deficiency anaemia with an Hb of 73 g/L. The patient wanted to have dental implants and during 2006 total parenteral nutrition (TPN) was given in preparation for surgery. In 2007, six fixtures were implanted in the maxilla.

In 2008, the patient underwent gastroscopy and colonoscopy which revealed two polyps just above the IRA; biopsies were taken and the pathological examination showed adenomas with low-grade dysplasia. Her biochemistry improved with a serum albumin of 33 g/L and an Hb within normal range, but in 2009, she suffered bilateral pitting oedema from time to time. Regastroscopy in 2010 revealed no polyps, ulcers, or blood but the colonoscopy showed multiple polyps below, at and above the IRA. Rectal biopsies showed adenomas with dysplasia and ileal biopsies revealed chronic inflammation.

**Figure 1:**
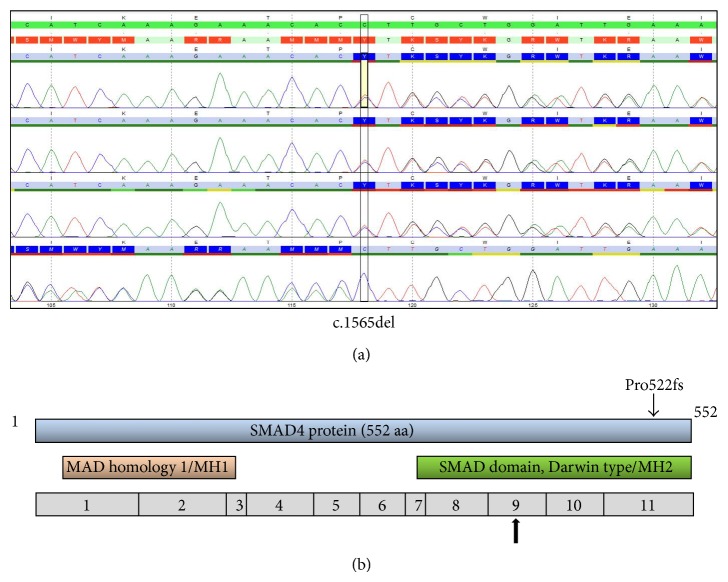
(a) Electropherogram of the mutation in* SMAD4*. (b) The SMAD protein and its domains. The novel mutation is indicated. At the bottom of the figure, the 11 exons in relation to the protein are shown. Early described mutational hot spot in exon 9 is marked out [6].

A year later, the patient presented with epigastric pains and the diagnosis gallstone pancreatitis was made. During the diagnostic workup, an ovarian cyst was found. Both disorders were treated with open surgery in one séance during which a vaginal polyp was found. Pathology revealed the cyst to be a cystadenofibroma and the polyp to be fibroepithelial. Her attending surgeon actualized her still undiagnosed syndrome suspecting some genetic disorder. A care conference was organized, responsibility for investigation and follow-up were distributed between professionals, and the geneticist suspected Juvenile polyposis syndrome in both the patient and her daughter. The patient underwent genetic screening for the genes* APC and MUTYH*, which was negative excluding the diagnosis of Familial Adenomatous Polyposis. Histological reevaluation of previous pathological samples did not show any sign of juvenile polyps. However, due to the clinical picture screening of the juvenile polyposis genes* SMAD4* and* BMPR1A* was performed on DNA extracted from peripheral blood leukocytes, with Sanger sequencing, including all coding exons and surrounding splice sites (−20 to +20). MLPA for deletion/duplication analysis was also performed (MRC-Holland, kit P158-C1). This revealed a novel, disease-causing mutation in* SMAD4* (c.1565delC, p.Pro522fs) (Figure 1(a)). The SMAD protein including mutation location is seen in Figure 1(b). The patient's pathogenic mutation was found 32 years after she presented with her first symptoms.

The mutation is a frameshift mutation in the highly conserved C-terminus and results in a truncated protein 17 amino acids prematurely. This alters the binding affinity of the protein, which may affect the homo/heterotrimeric interactions that* SMAD4* is involved in.

**Figure 2:**
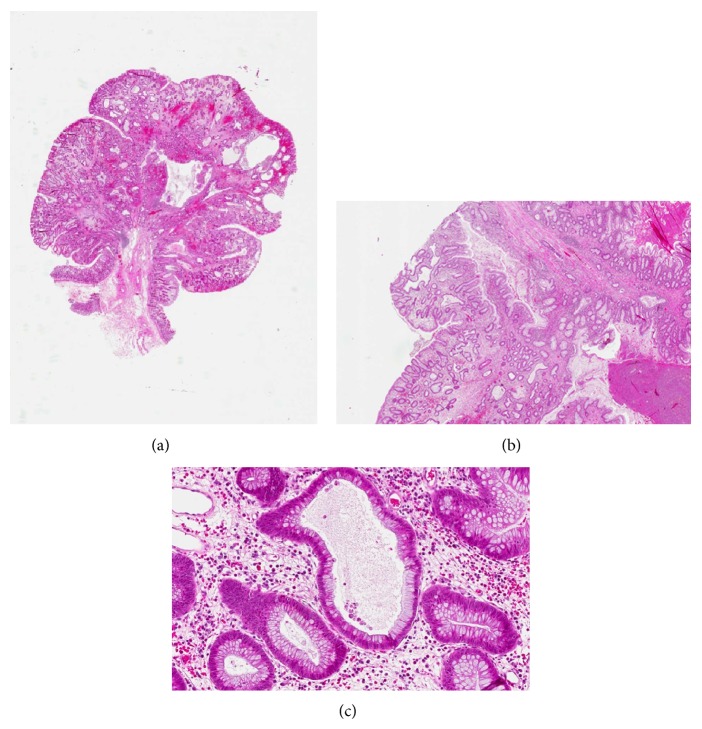
(a) Juvenile polyp surface with tortious glands of varying size focally filled with mucus and lined by columnar epithelium. Note the oedematous and inflamed stroma. (b) No smooth muscle fibres are detected. An oedematous and inflamed stroma with collagen fibers. (c) High magnification of part of the juvenile polyp with marked congestion and dilated glands with prominent mucin and cell debris. Infiltration of lamina propria with large amounts of mononuclear cells mainly plasma cells.

**Figure 3:**
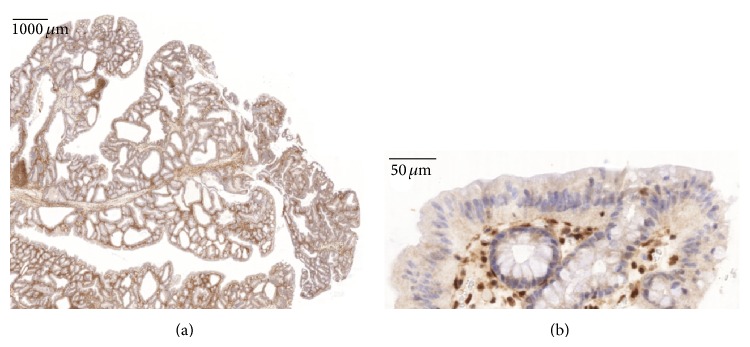
*SMAD4* immunohistochemistry. Loss of* SMAD4* expression in epithelial cells of the mucosa with retained expression in the stromal compartment. (a) Overview. (b) High power.

**Figure 4:**
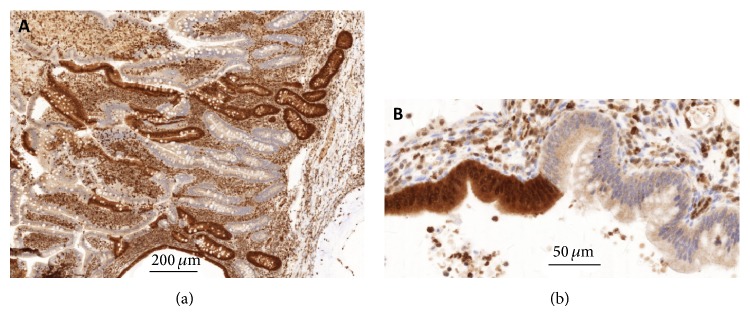
*SMAD4* immunohistochemistry. Loss of* SMAD4* expression in subset of epithelial cells of the mucosa. (a) Intermingled glands. (b) High power of transition within a gland.

Histological reexamination of a rectal polyp extirpated in 2011 showed a juvenile polyp (Figures 2(a)–2(c)). Immunohistochemical staining (using the monoclonal antibody sc-7966, Santa Cruz) reveals a total loss of* SMAD4* expression within the epithelial cells of the mucosa (Figures 2(a)-2(b)). When staining a polyp extirpated in 2014 loss of* SMAD4* expression was noted in most epithelial cells. However, glands positive and negative for* SMAD4* are in some areas intermingled (Figures 3(a)-3(b)) and examples of neighbouring cells within the same gland with and without* SMAD4* expression can be seen (Figures 4(a)–4(b)).

In early 2012, an abdominal ultrasound showed a 3 cm large polyp in the patient's small bowel, and a polyp resection through enterotomy was planned but cancelled due to a finding of hypoalbuminemia that proved to be resistant to TPN. Protein profile showed an IgG of 1.8 g/L and a repeat measure four months later showed an IgG of 2.4 g/L. At this point, her albumin had normalized and an operation was scheduled for after the summer, but albumin levels fell later that year and the operation was postponed. Subcutaneous gamma globulin was also tried, but the patient responded with fever and influenza-like symptoms and refused more treatment despite of a very low IgG level of 1.6 g/L.

Due to the association between* SMAD4* mutations and hereditary haemorrhagic telangiectasia, the patient was referred to echocardiography and ultrasound of the liver. As she had previously refused MRI no referral was sent for MRI head and neck.

After several delays due to hypoalbuminemia, the patient finally underwent a planned enterotomy with enteroscopy in the summer of 2014. Six polyps of varying size were resected from the Roux-en-Y area as well as a larger polyp in the distal duodenum. The pathology report showed juvenile polyps. The patient made an uneventful recovery and at follow-up two months later the patient's albumin was 38 g/L and IgG was 6.9 g/L.

## 3. Discussion

This case demonstrates how difficult it is to correctly diagnose polyposis disease and the severity of both disease and medical interventions, especially if care is fragmented. Today we are more aware of the complexity of the polyposis syndromes and that both clinical and genetic findings are important in the management of patients [7]. This case illustrates that providing care for patients with severe symptomatology, often because of rare diseases, is complex and involves a great number of medical specialties. We underline the importance and need for patients with rare disease to be managed within multidisciplinary teams together with representatives from the clinical genetics team [8]. This approach reduces the risk of stagnant clinical investigations and physicians loosing track of investigations. A multidisciplinary approach saves health care resources and reduces personal suffering. It is the ongoing work in several regions in Sweden to investigate the best approach for such care coordination. In the Southeast Healthcare region, this is performed within the Rare Disease project in accordance with the EU commission's recommendations [9, 10].

The present pathogenetic germline mutation of* SMAD4* fits well with the loss or partial loss of protein expression. Langeveld et al. have previously described the same findings and a correlation with somatic inactivation of* SMAD4 *[11].

This patient suffered multiple infections which made certain therapeutic approaches unfeasible, such as long-term intravascular catheters. The reason for this may be general malnutrition due to her short bowel and lack of appetite. However, her low albumin levels seem somewhat resistant to TPN and no other cause can be discerned, suggesting that the patient has protein loosing enteropathy, a known comorbidity to JPS [4]. This, together with the patient's low IgG, points towards loss of immunoglobulins as well as smaller proteins in the gastrointestinal tract causing the immunodeficiency of the patient. To our knowledge, this is the first reported case of immunodeficiency due to protein loosing enteropathy in JPS. The protein loosing enteropathy and immunodeficiency responded very well to resection of the polyps, implicating that they were the source of protein loss. Recurring haemorrhages has also been a running point in the management of this patient. The bleeding tendency is thought to be due to HHT. Gallione et al. have described the overlapping spectra of* SMAD4* mutations in JPS and HHT and underlined the importance of HHT evaluation in all patients with* SMAD4* mutations [12]. This patient only fulfils one of Curaçao criteria, repeated epistaxis, but due to the patient's long history of recurrent mucosal bleeding, where no other cause has been found, we strongly suspect HHT. The diagnostic workup has however not been completed respecting the patient's wishes.

## 4. Conclusion

Accurate diagnosis is essential for proper medical care. Patients with complex and severe symptomatology often have rare disorders that require care coordination, which is best provided by a multidisciplinary team including representatives from clinical genetics. Patients with* SMAD4* mutations should be followed up for both JPS and HHT, and physicians should be made aware of protein loosing enteropathy and immunodeficiency in JPS.
